# Reverse signaling via PD-L1 supports malignant cell growth and survival in classical Hodgkin lymphoma

**DOI:** 10.1038/s41408-019-0185-9

**Published:** 2019-02-19

**Authors:** Shahrzad Jalali, Tammy Price-Troska, Cole Bothun, Jose Villasboas, Hyo-Jin Kim, Zhi-Zhang Yang, Anne J. Novak, Haidong Dong, Stephen M. Ansell

**Affiliations:** 1 Division of Hematology and Internal Medicine, Mayo Clinic, Rochester, MN USA; 2 Department of Immunology, College of Medicine, Mayo Clinic, Rochester, MN USA

## Abstract

Treatment with programmed death-1 (PD-1) blocking antibodies results in high overall response rates in refractory and relapsed classical Hodgkin lymphoma (cHL) patients, indicating that PD-1/PD-1 ligand interactions are integral to progression of this disease. Given the genetically driven increased PD-L1/2 expression in HL, we hypothesized that reverse signaling through PD-1 ligands may be a potential mechanism contributing to the growth and survival of Hodgkin Reed–Sternberg (HRS) cells in cHL. Our data show that engagement of PD-L1 using an agonistic monoclonal antibody increases cell survival and proliferation and reduces apoptosis in HL cell lines. We show that HL patients have significantly higher serum levels of soluble PD-1 than healthy controls, and find that both membrane-bound and soluble forms of PD-1 are able to induce PD-L1 reverse signaling in HL cell lines. PD-L1 signaling, which is associated with activation of the MAPK pathway and increased mitochondrial oxygen consumption, is reversed by PD-1 blockade. In summary, our data identify inhibition of reverse signaling through PD-L1 as an additional mechanism that accounts for clinical responses to PD-1 blockade in cHL.

## Introduction

The advent of immunotherapy targeting immune checkpoint molecules has been associated with significant improvements in the treatment of several neoplasms, including hematological malignancies^1^. Programmed death-1 (PD-1) and its two cognate ligands, PD-L1 and PD-L2, are immune modulatory molecules that are expressed on both hematopoietic and non-hematopoietic cells and are involved in maintaining immune homeostasis. While the interaction of PD-1 with its ligands is necessary for immune tolerance, it can provide a mechanism for cancer cells to escape from immune surveillance. In fact, increased expression of PD-1 ligands by cancer cells, arising from either genetic alteration or microenvironmental triggers, and their binding to PD-1 receptors on the surface of T cells has been shown to attenuate T-cell receptor (TCR)-mediated signaling and result in an exhausted T-cell phenotype that can prevent lysis of tumor cells^2,3^.

Classical Hodgkin lymphoma (cHL) is a B-cell malignancy that is characterized by the presence of a small number (1–5%) of Hodgkin Reed–Sternberg (HRS) cells surrounded by an extensive infiltration of various immune cell types that comprise more than 90% of the cells within the tumor lesion. Analysis of the immune cells has identified CD4 + T cells as the predominant cell population within tumor microenvironment in cHL. The CD4^+^ T-cell population contains PD-1 + Th1-polarized, rather than Th2-polarized, effector T cells and also PD-1-negative regulatory T cells^4–7^, implying an immunosuppressive microenvironment. PD-1 + CD4 + T cells, together with tumor-associated macrophages (TAMs) are located in close proximity to HRS cells, comprising a unique niche in cHL^8^.

Overexpression of PD-L1 and PD-L2, driven by genetic alterations and deregulated signaling pathways, has been identified in HRS cells and mediates immune evasion by HRS cells. Amplification or copy number gain of chromosome 9p24.1 has been identified in almost all cHL patients and has shown to be associated with increased transcript levels of PD-1 ligands in both cHL cell lines and primary HRS cells^9^. Elevated levels of PD-L1 are also observed in cHL with normal or low 9p24.1 amplification, an effect that is regulated by AP-1 activation and EBV infection^10^. The increased expression of PD-1 ligands is predicted to induce immune suppression upon engagement of PD-1 receptors on effector T-cells, thereby creating a strong rationale for blocking PD-1 signaling to clinically benefit patients with cHL. Clinical use of anti-PD-1 antibodies has resulted in response rates of 65–87% in relapsed or refractory HL patients^11–13^, implying that the blockade of PD-1/PD-L1 or -L2 signaling could trigger a T-cell-mediated immune response against tumor neoantigens. However, lack or reduced HRS cell surface expression of β_2_-microglobulin, MHC class I, and MHC class II complex, which are seen in 80%, 78%, and 67% of the cHL patients, respectively^14^, restricts antigen presentation and effector T-cell function suggesting that other mechanisms may be relevant.

Recent results have shown that genetically driven PD-L1 expression and MHC class II positivity on HRS cells in cHL, rather than MHC class I expression, are potential predictors of favorable outcome after PD-1 blockade^15^. While this suggests a CD4 + T cell-mediated mechanism of response, a subset of patients with MHC class II-negative HRS cells also responded to PD-1 blockade, suggesting that additional mechanisms may play a role. Owing to the genetically driven PD-L1 amplification in HRS cells and the association of PD-L1 expression with response to PD-1 blockade, we explored the role of PD-L1 reverse signaling in the context of immune checkpoint inhibition in cHL.

## Results

### PD-L1 reverse signaling increases survival and proliferation of the HL cell lines

**Fig. 1:**
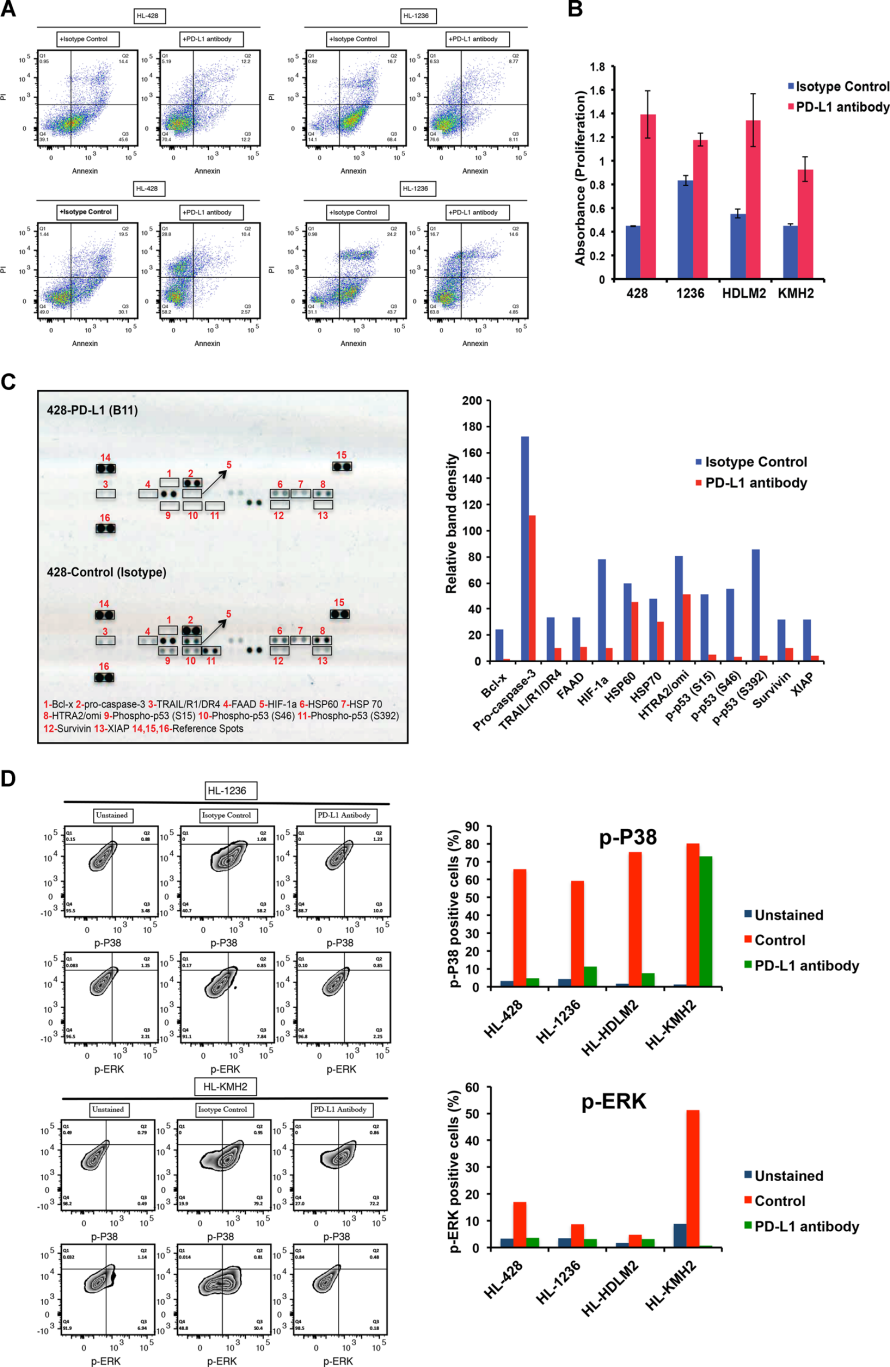
Stimulation of the HL cell lines with PD-L1 antibody increases cell survival and proliferation and reduces apoptosis. HL cell lines (HL-428, HL-1236, HL-HDLM2, and HL-KMH2) were starved overnight and plated over either isotype control (IgG1) antibody- or PD-L1 antibody-coated (10 μg/ml) plates for 48 h. **a** Flow-cytometry analysis showing Annexin/PI staining on four different HL cell lines. **b** Bar graphs showing the proliferation rate of HL cell lines, in response to incubation with isotype control (blue) or PD-L1 antibody (red). **c** Apoptotic protein array illustrating the differences between apoptotic proteins and phospho-proteins in the HL-428 cells that are incubated with isotype control or PD-L1 antibody (left panel). Bar graphs show the quantitative analysis of the protein dots using ImageJ (right). **d** Flow-cytometry analysis (left) displays the intracellular staining with p-ERK and p-P38 antibodies in two HL cell lines incubated with isotype control or PD-L1 antibodies. Bar graphs (right) represent the percentage of the cells that are positive for p-P38 and p-ERK in four different cell lines

HL cells express elevated levels of PD-L1 as a result of either chromosome 9p24.1 amplification or EBV infection. While the interaction of PD-L1 with its receptor PD-1 could suppress T-cell function, the reverse effect of such an interaction on the HL cells has not been elucidated. We used an agonistic mouse monoclonal antibody targeting PD-L1^16^ (provided by Dr. Dong) to stimulate PD-L1 on the cell surface of HL cell lines to study the reverse signaling through PD-L1. Using flow-cytometry analysis, we first examined the expression of PD-L1 by all four HL cell lines (HL-428, HL-1236, HL-HDLM2, and HL-KMH2) used in this study. Our data showed PD-L1 surface expression on all cell lines. Mean fluorescent intensities (MFI) were reported as: HL-428 (Isotype control: 623, PD-L1: 967), HL-1236 (Isotype control: 1522, PD-L1: 8270), HL-HDLM2 (Isotype control: 492, PD-L1: 5432), and HL-KMH2 (Isotype control: 297, PD-L1: 532) (Supplementary Fig. 1A). To ensure that it is the PD-L1 that transmits the signals and no other ancillary receptor is involved, the four different HL cell lines were starved overnight and then seeded over the plates coated with 10 μg/ml of either IgG1 isotype (control) or PD-L1 antibody in the starvation culture medium. As a result of starvation for 2 days, control cells underwent apoptosis and cell death (Fig. 1a), while cells incubated with the PD-L1 antibody showed a considerably reduced level of both apoptosis and cell death (Fig. 1a), indicating that the incoming signal from PD-L1 significantly promoted the survival of the tumor cells. To further confirm the engagement of PD-L1 by the PD-L1 agonistic antibody, we treated HL cells (either the parent line or PD-L1 overexpressing cells) with a PD-L1-blocking antibody prior to seeding them on the PD-L1 agonistic antibody-coated plates. Our data showed a shift toward increased apoptosis in this group of the cells, verifying that reverse signaling via PD-L1 enhances cell survival in HL cells (Supplementary Fig. 1B). We then examined the effect of the PD-L1 antibody on HL cell proliferation. As shown in Fig. 1B, HL cells treated with the PD-L1 antibody had significantly increased proliferation when compared with control cells.

We next utilized an array-based analysis to identify the proteins that are involved in mediating increased survival of HL cells due to the PD-L1 reverse signaling. Our data using HL-428 cells identified several apoptotic proteins and phosphoproteins, which were highly downregulated in the presence of PD-L1 antibody, including Bcl-x, procaspase 3, XIAP, survivin, and phospho-p53 (Fig. 1c). Furthermore, p53 also had a role in the anti-apoptotic effect induced by PD-L1 reverse signaling, as expression of phospho-p53 proteins were profoundly reduced in this analysis (Fig. 1c).

### PD-L1 reverse signaling is associated with the MAPK pathway in HL tumor cells

A previous study has shown that reverse signaling through PD-L1 can occur in cytotoxic T cells and that engagement of PD-L1 by an agonistic antibody increases phosphorylation of P38^16^. To determine whether increased survival and proliferation of the HL cells in response to the PD-L1 antibody is linked to the MAPK signaling molecules, we tested the phosphorylation of both P38- and ERK–MAPK (p-P38 and p-ERK) in the control IgG1 isotype or anti-PD-L1-treated HL lines. As shown in Fig. 1d, in response to the agonistic anti-PD-L1 antibody, both p-P38 and p-ERK decreased in all HL lines, consistent with previous data showing that decreased phosphorylation of P38 provides a pro-survival signal^16^. This implied that signaling through PD-L1 employs the MAPK pathway and that there is an inverse correlation between cell survival and phospho-MAPK levels.

### Treatment with nivolumab reverses the PD-L1 reverse signaling phenotype induced by PD-1/PD-L1 interaction

**Fig. 2:**
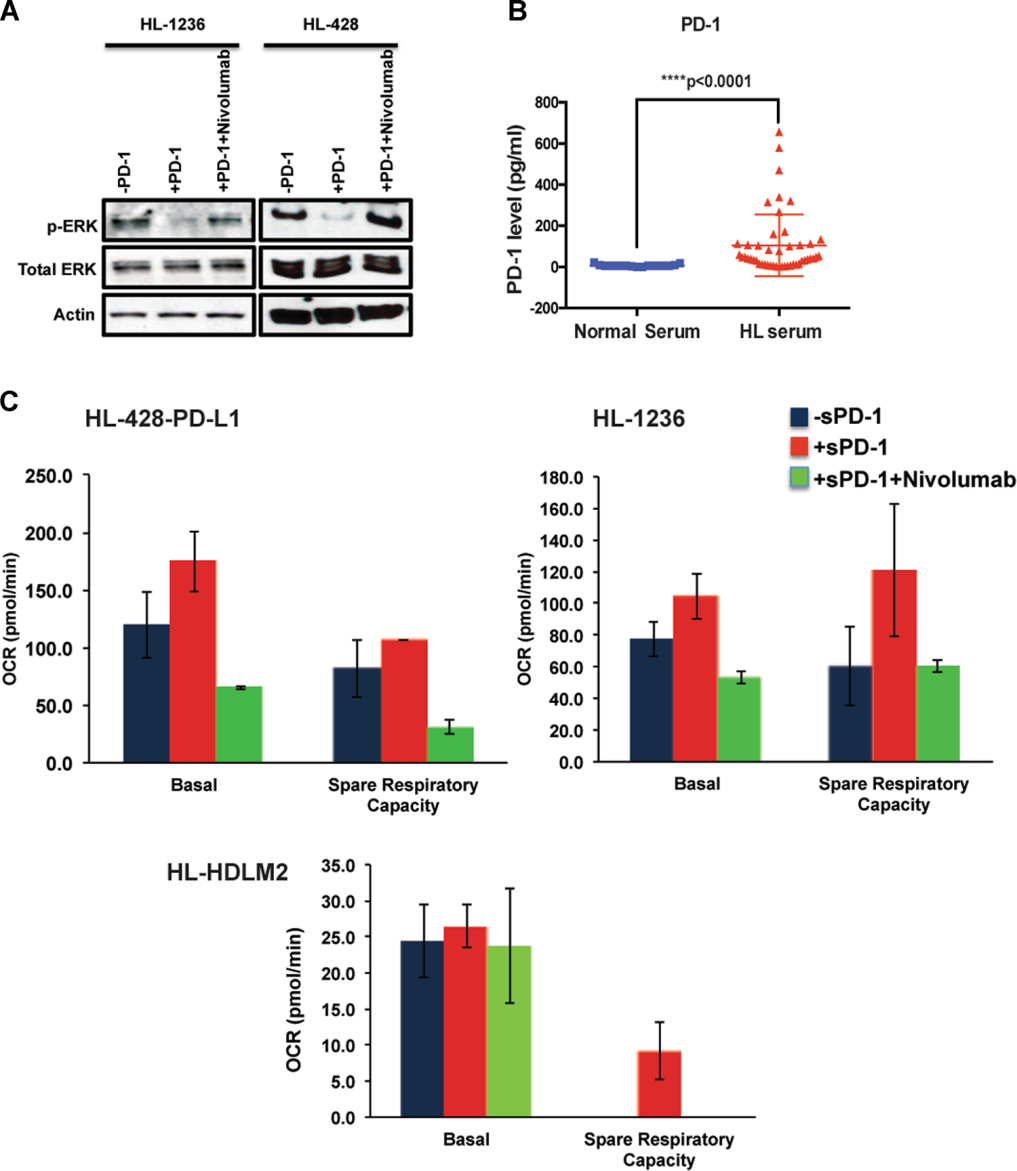
Both membrane-bound and soluble forms of PD-1 induce the PD-L1 reverse signaling and nivolumab treatment counteracts this effect in HL cells. **a** Effect of membrane-bound PD-1 on ERK phosphorylation. Dox-inducible PD-1 expressing HEK293 cells were treated with Dox (2 μg/ml) for 48 h and then cocultured with starved GFP + HL-428 or HL-1236 cells for 24 h. Cells were sorted and GFP + cells were collected, followed by western blot analysis using p-ERK and total ERK antibodies. Actin staining shows the protein loading control. **b** Soluble PD-1 is increased in the serum of the HL patients. Serum samples were obtained from HL patients and normal donors and used to determine the concentration of soluble PD-1. As shown in the dot plot, the concentration of soluble PD-1 is significantly higher in HL patients than normal samples (****p < 0.0001, normal serum: *n* = 17; HL serum: *n* = 47). **c** Effect of soluble PD-1 on mitochondrial respiration. HL cell lines were starved overnight and then incubated with recombinant Fc-chimera active PD-1 protein (10 μg/ml) in the presence r absence of Nivolumab. An equal number of the cells were plated on the microplates and used for mitochondrial stress analysis using XFe96 seahorse analyzer and data were analyzed using Wave software. The plot represents oxygen consumption rate (OCR) at baseline and over time following oligomycin, FCCP, and rotenone injection in the HL-1236 cell line. Bar graphs show basal OCR and spare respiratory capacity in three different HL lines

We hypothesized that interactions between PD-1 ligands and the PD-1 receptor would provide a similar signal to that seen when the agonistic anti-PD-L1 antibody was used. We first tested the stimulatory effects of membrane-bound PD-1 and evaluated the effect of the blocking antibody nivolumab in counteracting such effects. We generated doxycycline-inducible PD-1 overexpressing HEK293 cells to be used as the membrane-bound receptor for PD-L1. Coculture of the HL-428 and HL-1236 cells with PD-1-expressing HEK293 cells resulted in reduced p-ERK protein in HL cells, and treatment with nivolumab reversed this effect and increased p-ERK to normal levels (Fig. 2a). Given that reduced phosphorylation of ERK and P38 is associated with a pro-survival signal^16^ (Fig. 1d), these data were consistent with the flow-cytometry results showing reduced p-ERK levels in response to incubation with the agonistic anti-PD-L1 antibody (Fig. 1d), and confirms the fact that PD-L1 reverse signaling also occurs as a consequence of PD-L1/PD-1 interaction. Furthermore, treatment with an anti-PD-1 antibody, nivolumab, counteracts this signaling process.

### Hodgkin lymphoma patients have elevated circulating levels of soluble PD-1

Owing to the modest expression of PD-1 seen in cHL patient biopsies, we tested whether soluble PD-1 was increased in patient sera and could provide a similar agonistic signal. Because, increased levels of soluble immune checkpoint molecules including PD-1 have been shown to be a prognostic factor in several malignancies^17–20^, we first measured the soluble form of PD-1 in the serum of cHL patients and compared the levels to those seen in matched normal controls. As shown in Fig. 2b, soluble PD-1 concentrations were significantly higher (*p* < 0.0001) in the patient’s serum than normal subjects, suggesting a potential biological significance of this molecule in the peripheral circulation.

In a separate set of the experiments, we utilized a recombinant PD-1 protein (Fc Chimera), as the soluble receptor and addressed the function of soluble PD-1 in inducing PD-L1 reverse signaling. Given that proliferating malignant cells have increased energy demand and interfering with this process could prevent malignant cell growth and survival, we treated HL cells with soluble PD-1 and examined the oxygen consumption rate (OCR) of HL cell lines, as a measure of mitochondrial respiration and metabolic status of the malignant cells. Except for HL-428-PD-L1, basal OCR level did not differ between different treatment groups in HL-1236 and HL-HDLM2. However, spare respiratory capacity of all three HL lines was increased in response to soluble PD-1 and addition of the anti-PD-1-blocking antibody nivolumab reversed this effect (Fig. 2c). Altogether, these findings indicate that the anti-PD-1 antibody nivolumab can prevent the interaction of PD-L1 with both membrane-bound and soluble forms of PD-1, and thus inhibit PD-L1-mediated signaling in HL cells that promotes the metabolic activity of tumor cells.

## Discussion

Immune checkpoint blockade has shown significant clinical efficacy in the treatment of several cancers, including non-small-cell lung cancer^21^, ovarian cancer^22^, metastatic melanoma^23^, and hematologic malignancies^1^. In Hodgkin lymphoma, treatment with PD-1 inhibitors has been associated with high objective response rates in the patients with refractory or relapsed disease^11–13^. Amplification of chromosome 9p24.1^9^ and activation of AP-1^10^ by HRS cells result in increased expression of PD-L1 and/or PD-L2, and interaction of these ligands with PD-1 + effector T cells inhibits an effective anti-tumor immune response. Therefore, the underlying mechanism of therapeutic response to PD-1 blockade in Hodgkin lymphoma could be explained by the fact that the disruption of PD-1/PD-L1 or PD-L2 interaction initiates a cascade of signaling events that reverses the exhausted phenotype in T cells and triggers an effective immune cell response against tumor cells^24^. In fact, a recent study indicated that the total number of T cells, as well as the CD4 and CD8 populations, are increased in the peripheral blood of HL patients who are treated with a PD-1-blocking antibody, supporting the notion that the T-cell-mediated response^12^ could be a major mechanism accounting for the efficacy of anti-PD-1 antibodies.

However, the loss or reduced expression of MHC class I and II molecules on HRS cells^14^ would be expected to limit neoantigen presentation and subsequent effector T-cell function, suggesting that additional and/or alternate mechanisms may contribute to the response to PD-1 blockade therapy in HL. A recent report has shown that TAMs constitute the majority of PD-L1 + cells within the tumor microenvironment in HL. These cells are in close vicinity of PD-L1 + HRS cells and engage a large number of the PD-1 + T cells, thereby generating an immunosuppressive niche in cHL^8^. The PD-1 + T cells predominantly exhibit a CD4 + Th1-polarized T-effector phenotype and are expected to be able to lyse HRS cells when immune checkpoint blockade is used^6^. Whether it is TAMs, T cells or a combination of both cell types that promote the anti-tumor immunity following PD-1 blockade has yet to be determined. While T cells and TAMs are shown to be prevalent in the tumor microenvironment, an increased number of natural killer cells are also found in the peripheral blood circulation, and these cells may be recruited to the tumor and contribute to the lysis of MHC-deficient malignant cells^12^.

While immune mechanisms may account in part for the efficacy of PD-1 blockade in cHL, we explored whether genetic reasons for PD-1 ligand overexpression provided a growth advantage to HRS cells and whether engagement of the ligands with PD-1 activated the tumor cells directly. Herein, we confirm the presence of reverse signaling through PD-L1 in the HL cell lines and explored its role in response to anti-PD-1 therapy. We describe that the engagement of PD-L1 using a mouse anti-human agonistic PD-L1 monoclonal antibody (clone B11, in-house generated by Dr. Dong lab) increases the survival of HRS cells by significantly reducing the number apoptotic and dead cells in all four HL cell lines examined in this study (Fig. 1a). Moreover, the agonistic anti-PD-L1 antibody increases the cell proliferation rate (Fig. 1b) and reduces the protein expression of several apoptotic proteins (Fig. 1c), indicating that the increased expression of PD-L1 on the cell surface of HL cells promotes proliferation and survival of the tumor cells. In support of these findings, upregulation of PD-L1 has been shown to promote cell proliferation in pancreatic cancer cells^25^, a finding we also confirmed by overexpressing PD-L1 using a retroviral system and noting an increase in the proliferation rate of HL lines (data not shown). Similarly, others have shown that PD-L1 cell-intrinsic signaling protects tumor cells from interferon-induced cytotoxicity via a non-conventional conserved motif located within the intracellular domain of PD-L1 and leads to tumor progression in both murine and human cell lines^26^. While this study indicates that blocking of PD-L1 receptor sensitizes tumor cells to interferon-induced cytotoxicity, no data are available, testing whether PD-1 blockers would induce similar sensitization in HRS cells.

In this report, we show that treatment of HL cells with an agonistic PD-L1 antibody reduces the level of p-P38 and p-ERK (Fig. 1d). In addition, membrane-bound PD-1 also triggered reverse signaling through PD-L1 (Fig. 2a) and treatment with nivolumab inhibited this effect, suggesting that clinical responses in cHL patients to PD-1-blocking antibodies are, at least in part, mediated by interrupting PD-L1 signaling. It has previously been reported that the PD-L1 antibodies capable of increasing p-P38 level induce the apoptosis of tumor-infiltrated cytotoxic T cells^16^. Therefore, our observation of reduced p-P38 levels, decreased apoptosis, and an increased proliferation rate is consistent with this data and indicates that cell survival and proliferation and MAPK activity have an inverse correlation in HL cells. However, a previous study has suggested that cross-linking of PD-L1 using a PD-L1 monoclonal antibody (R&D Systems) induces cell apoptosis in EBV-transformed B cells^27^, a result that differs from our data that shows reduced apoptosis with an agonistic anti-PD-L1 antibody. The reason for this discrepancy may be explained by differences in antibody-binding site or cell type. However, the PD-L1 antibody we used (B11 clone) resulted in a similar phenotype as binding to PD-1, implying that PD-1 and B11 antibody share the same binding site on PD-L1. Future studies are required to address the different binding sites on PD-L1.

Our results further showed that the soluble form of PD-1 is significantly increased in the serum of HL patients when compared with normal subjects (Fig. 2b). The prognostic significance of soluble PD-1 and its ligands has previously been described in several malignancies, including melanoma, lung, and pancreatic cancers^17–20^, but this the first to report that soluble PD-1 is elevated in the circulation in HL patients. Although the biological properties of soluble PD-1 need to be precisely investigated, data presented in this study found that soluble PD-1 has the ability to induce PD-L1 reverse signaling in HL cell lines, similar to that seen with membrane-bound PD-1. In fact, treatment of the cells with soluble PD-1 increased spare respiratory capacity in three HL cell lines, and treatment with nivolumab could reverse this effect, suggesting that there is a functional and biological importance to these soluble forms (Fig. 2c). A recent report indicates that HRS energy metabolism is highly dependent on oxidative phosphorylation due to upregulation of several mitochondrial genes and increased mitochondria biogenesis as compared with germinal cell B-cells counterparts^28^. Similarly, another study using immunohistochemical analysis has shown that the markers of mitochondrial oxidative phosphorylation, such as TOMM20 and MCT1, are higher in HRS cells, but absent in TAM and tumor-associated lymphocytes. Instead, TAMs show increased reactivity for glycolysis marker MCT4 and are dependent on glycolysis, emphasizing the highly metabolic heterogeneity in cHL. According to this study, the high metabolic heterogeneity is present in the refractory and relapsed cHL (hazard ratio of 5.87)^29^. Therefore, the effects of PD-1 blockers on HL patients could be mediated by the disruption of HRS mitochondrial oxidative phosphorylation, and our data suggest that PD-L1 reverse signaling plays a significant role in this context.

In summary, our data identify a role for PD-L1 reverse signaling in promoting tumor cell growth, proliferation, and metabolism in cHL. We also find that interference with PD-L1 reverse signaling contributes to the therapeutic response seen with anti-PD-1-blocking antibodies in HL patients.

## Methods

### Cells and transfection

HL428, HL1236, HDLM2, and KMH2 cell lines were obtained from DSMZ (Leibniz-Institut, Germany). Cells were maintained in RPMI-1640 (Gibco), supplemented with 10% FBS (Gibco), 1% penicillin/streptomycin (Corning), 1 mM MEM nonessential amino acids (Corning), and 1× sodium pyruvate (Corning), at 37 °C incubator with 5% CO_2_. HEK293 cells were cultured in DMEM containing 10% FBS and 1% penicillin/streptomycin. The basal PD-L1 expression level in all four HL cell lines was determined using flow cytometry (Supplementary Fig. 1A).

To determine the role of genetically driven increased PD-L1 expression, HL cell lines were transfected with PD-L1-coding sequence using the pLEX-MCS Inducible Expression System (Open Biosystems). The gene of interest (GOI) was created by RT-PCR. Primers were designed to incorporate restrictions sites *XhoI* (5′) and *AgeI* (3′) followed by ligation into the pLEX-MCS vector using T4 DNA Ligase (New England Biolabs). DH5a (*E. coli*) competent cells were used to amplify lentivirus plasmid. The virus was produced according to the manufacturer instructions and introduced into HL cell lines. After 5 days of exposure to the virus, the transfected cells were selected using puromycin. Fourteen days post transfection, cells were screened for the expression of PD-L1 by flow cytometry (Supplementary Fig. 1A). MFI for PD-L1 overexpressing HL cells were: HL-428-PD-L1 (Isotype control: 20.4, PD-L1: 1576), HL-1236-PD-L1 (Isotype control: 48, PD-L1: 948), and HL-KMH2-PD-L1 (Isotype control: 35.9, PD-L1: 1496).

HEK293 cells were transfected with PD-1-coding sequence using the Retro-X™ Tet-On^®^ 3 G Inducible Expression System (Takara Bio USA, Mountainview, CA). The retrovirus was produced according to the manufacturer instructions and introduced into HEK293 cell line. After 5 days of exposure to the virus, the cells were selected using puromycin. After 1 week of selection, the cells were exposed to doxycycline. After 5 days, the cells were screened for expression of PD-1 by FACS, western, and immunohistochemistry.

### Luminex assay

Serum samples from 47 Hodgkin lymphoma patients were collected to test the presence of soluble PD1, and 17 normal serum samples were added as a control. The samples were analyzed in a multiplex array using ProcartaPlex magnetic beads on a Luminex^®^ platform (Austin, Tx). The individual bead, Human PD-1 (CD279) (cat#EPX01A-12214-901), was purchased from ThermoFisher Scientific (Waltham, MA). All other reagents were supplied by the ProcartaPlex Human Basic Kit (cat#EPX010-10420-901, ThermoFisher Scientific). The bead mix was prepared according to the manufacturer’s protocol. All serum samples were run in duplicate. Each well in the 96-well plate contained 25 µl of Universal Assay Buffer and 25 µl of patient serum, then analyzed using the Luminex^®^ 200™ instrument. The data were analyzed using the instrument’s software xPONENT.

### Apoptosis analysis

To determine the effect of PD-L1 ligation on apoptosis, 24-well plates were coated with 10 μg/ml of IgG1 isotype control (Sigma) or an agonistic monoclonal PD-L1 antibody^16^ (generously provided by Dr. Dong) overnight at 4 °C. HL428 cell lines were starved overnight and incubated on the coated plates in the starvation medium (RPMI containing 0.5% BSA). Following 48 h, cells were washed with Annexin-V binding buffer (10 mM HEPES, 150 mM NaCl, and 3 mM CaCl_2_; pH 7.4) and stained with 5 μl Annexin-V (Fluorescein-conjugate, from Invitrogen) for 30 min and then with 0.5 μg/ml of propidium iodide (PI) (Sigma Aldrich) for 5 min. A minimum of 10,000 cells stained with Annexin/PI were analyzed using on a BECTON DICKINSON (BD) FACSCANTO II and data were processed by FlowJo software (V10.4).

Relative levels of apoptotic-related proteins were determined using Human Apoptosis Array Kit (R&D Systems), according to the manufacturer’s instructions. Briefly, HL428 cells were starved overnight and then incubated over IgG1 isotype control (Sigma) or PD-L1 antibody-coated plate for 24 h. Cells were then washed in PBS and lysed with cell lysis buffer supplemented with complete protease inhibitor cocktail (Roche) and halt phosphatase inhibitor cocktail (Thermo Scientific). In total, 300 μg of total proteins were incubated with the nitrocellulose membranes containing 35 different capture antibodies printed in duplicates, overnight at 4 °C. The membranes were then washed and incubated with detection antibody cocktail for 1 h at room temperature. The blots were incubated with streptavidin–HRP and Chemi reagents, respectively, and exposed to X-ray film to detect the protein signals. After scanning the developed X-ray films, the pixel densities were determined using ImageJ analysis software.

### Oxygen consumption rate (OCR) measurement

To assess the effect of PD-L1 blockade on mitochondrial respiration of the cells, parent line and PD-L1 overexpressing HL cells were starved overnight and then incubated with the growth medium containing 10 μg/ml recombinant human PD-1 protein (Active Fc Chimera from Abcam) in the presence or absence of 10 μg/ml anti-PD-1 (nivolumab, Bristol-Myers Squibb). Following 3 days, cells were analyzed for OCR using Cell Mito Stress kit (Agilent) and the Seahorse XFe96 Extracellular Flux Analyzer (Seahorse Bioscience). The optimal cell density and Carbonyl cyanide-4 phenylhydrazone (FCCP) concentration were determined using XF Cell Energy Phenotype Test kit. Briefly, HL cells were resuspended in Agilent Seahorse XF Base Medium RPMI without phenol red, plated onto Seahorse cells plates (3 × 10^4^–5 × 10^4^) and incubated in CO_2_-free humidified 37 °C incubator for 1 h. Mitochondrial respiration function was measured at basal level, which represents the energetic need of a cell under baseline condition. In addition, spare respiratory capacity, an indicative of maximum capacity or flexibility of a cell to respond to an energetic demand, was assessed by sequential injection of electron transport chain modulators, including 1 μM oligomycin, 1 μM carbonyl cyanide-4 phenylhydrazone (FCCP), and 1 μM rotenone/antimycin A, over an 80-min period. The analyzed stress test parameters were exported from the analyzer, and the graphs were prepared by GraphPad Prism (V6) software.

### Flow cytometry

Starved HL cells were seeded over isotype control or PD-L1 antibody-coated plates as described above. Forty-eight hours later, cells were in 4% formaldehyde for 10 min at 37 °C and then permeabilized in ice-cold methanol for 30 min on ice. Cells were then washed and incubated with rabbit mAb to phospho-p38 MAPK (Thr180/Tyr182) (Clone D3F9, Cell Signaling), rabbit mAB to phospho-p44/42 MAPK (Erk1/2) (Thr202/Tyr204) (D13.14.4E, Cell Signaling) or Isotype control IgG for 1 h at room temperature, followed with incubation with secondary Alexa Fluor^®^ 488 AffiniPure F(ab’)_2_ fragment goat anti-rabbit IgG (H + L) (Jackson Immunoresearch Laboratories) for 30 min at room temperature. The intracellular levels of phospho-p38 and p-ERK MAPKs were analyzed using FACSCANTO II.

### Cell proliferation

Proliferating viable cells were determined using CellTiter 96^®^ AQueous One Solution Cell Proliferation Assay kit (Promega; cat#3580). Ninety-six well plates were first coated using isotype control or PD-L1 antibody as described in previous sections. Starved HL cell lines were seeded on the coated plated and incubated for additional 48 h. In total, 20 µl of CellTiter 96^®^ AQueous One Solution Reagent was then added to each well of the 96-well assay plate containing 100 µl of cell suspension in the culture medium. Cells were placed at 37 °C cell culture incubator for 2 h before the absorbance was measured at 490 nm using ELISA plate reader (spectraMax 190).

### Western blotting

Dox-induced PD-1 expressing HEK293 cells were cultured in the DMEM containing 10% FBS. Cells were either treated with 2 μg/ml Dox to express PD-1 or left untreated to serve as the control without PD-1 expression. Dox-induced PD-1 expression by HEK293 cells was confirmed by flow cytometry. HL-428 and HL-1236 cells expressing the green fluorescent protein (GFP) were starved and then cocultured with control or PD-1 expressing HEK293 cells, in the presence or absence of 10 μg/ml Nivolumab, for 24 h. GFP + cells were then collected by cell sorting and then lysed and used for western blot analysis using p-ERK, total ERK, and actin antibodies (Cell Signaling).

### Statistical analysis

Wilcoxon-paired non-parametric test was used to examine the significant differences between mean values of patients and normal serum samples. All analyses were performed on the GraphPad Prism (V6) software, and data were reported as mean ± SE.

## Supplementary information

supplementary Figure Legend.

Supplementary Fig.1.
