# Supplementary material for: Reverse signaling via PD-L1 supports malignant cell growth and survival in classical Hodgkin lymphoma
Source: Blood Cancer J. 2019 Feb 19;9(3):22. doi: 10.1038/s41408-019-0185-9 (PMC6381098; doi:10.1038/s41408-019-0185-9)
Supplement: Supplementary file 1 — supplementary Figure Legend. [file 41408_2019_185_MOESM1_ESM.docx]

### Supplementary Figure Legend.

### Supplementary Fig. 1.

### A. PD-L1 expression level in HL cell lines. Histograms represent flow cytometry analysis of basal and post-transfection level of PD-L1 expression by HL parental cell lines (upper panel) and HL cell lines transfected with PD-L1 (lower panel). Isotype control: Red; PD-L1; Blue. B. PD-L1 blocking antibody reverses the survival effect induced via PD-L1 on HL cell lines. Dot plots showing Annexin/PI staining using flow cytometry analysis. Starved HL-428, HL-428-PD-L1 and HL-KMH2-PD-L1 cell lines were cultured over the plates coated with isotype control or anti-PD-L1 agonistic antibody, in the presence or absence of PD-L1 blocking antibody, for 48 hours. Cells were then stained with Annexin/PI and analyzed by flow cytomtery.

### 
