# Supplementary figures and images for: Reverse signaling via PD-L1 supports malignant cell growth and survival in classical Hodgkin lymphoma
Source: Blood Cancer J. 2019 Feb 19;9(3):22. doi: 10.1038/s41408-019-0185-9 (PMC6381098; doi:10.1038/s41408-019-0185-9)

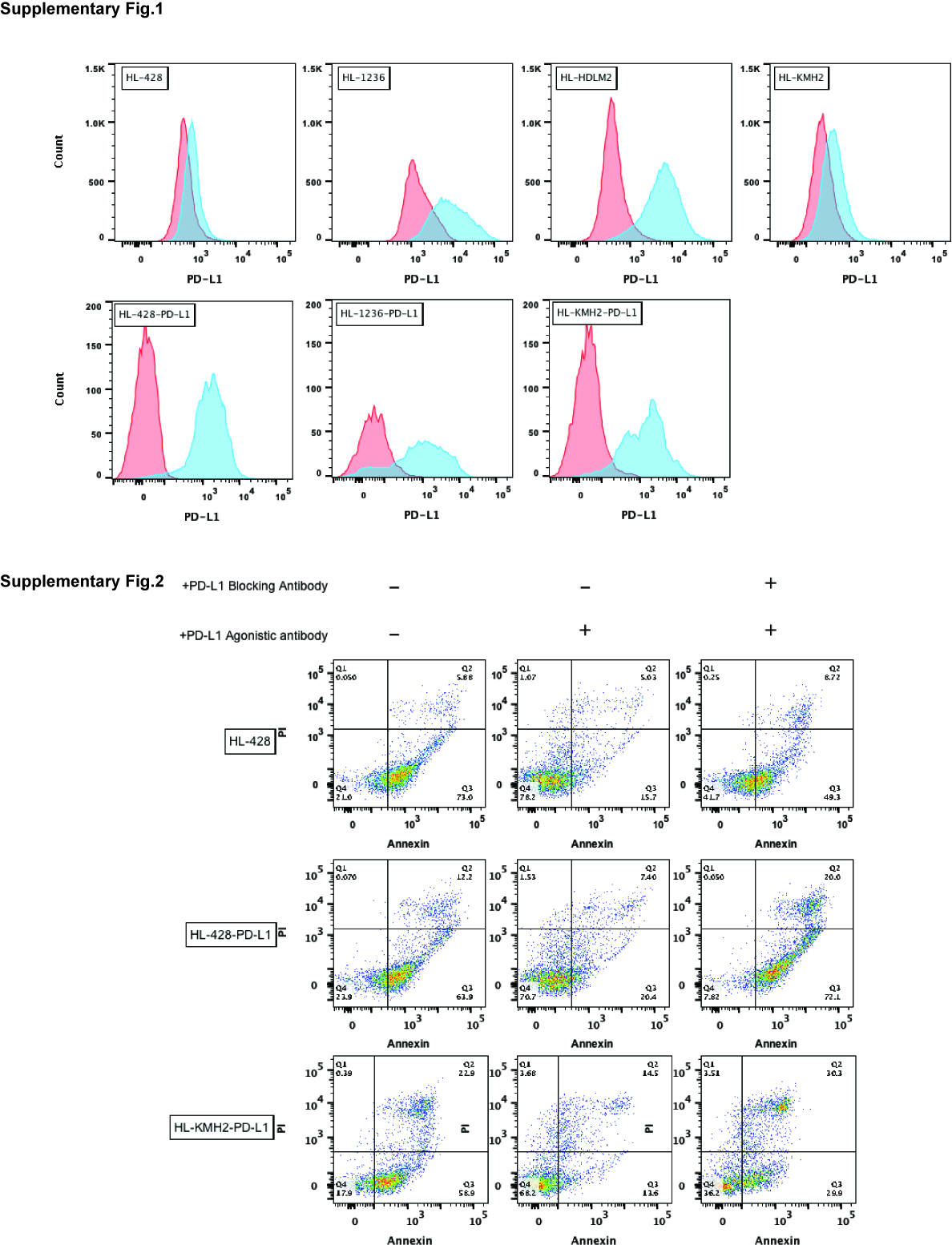

Supplement: Supplementary file 2 — Supplementary Fig.1. [file 41408_2019_185_MOESM2_ESM.tif]
